# Supplementary material for: Long term miscarriage-related hypertension and diabetes mellitus. Evidence from a United Kingdom population-based cohort study
Source: PLoS One. 2022 Jan 21;17(1):e0261769. doi: 10.1371/journal.pone.0261769 (PMC8782476; doi:10.1371/journal.pone.0261769)
Supplement: S1 Table — (DOCX) [file pone.0261769.s001.docx]

**Supplementary File**

**S1 Table**. Search strategy based to Read codes.

The Read codes used to define the exposure, outcomes and covariates were generated after the following sequential steps; (i) Creation of a comprehensive list of search terms that denote a relevant diagnosis. (ii) A search of the Read code thesaurus using the list of search terms (iii) A search of additional codes from online Read code repositories or from supplemental information of published studies. [1,2] (iv) Rating each code for sensitivity and determining the final list by consulting clinical experts (general practitioners and consultant specialists)

Miscarriage Read codes.

|  |  |  |  |
| --- | --- | --- | --- |
| L04..00 | Spontaneous abortion | L041z11 | Retained products after spontaneous abortion |
| L04..11 | Miscarriage | L042.00 | Spontaneous abortion complete |
| L040.00 | Spontaneous abortion unspecified | L042000 | Complete spontaneous abortion + genital tract/pelvic infect |
| L040000 | Unspecified spontaneous abortion + genital tract/pelvic infection | L042100 | Complete spontaneous abortion +delayed/excessive hemorrhage |
| L040011 | Spontaneous abortion with sepsis | L042200 | Complete spontaneous abortion + pelvic organ/tissue damage |
| L040100 | Unspecified spontaneous abortion + delayed/excessive hemorrhage | L042300 | Complete spontaneous abortion with renal failure |
| L040111 | Spontaneous abortion with heavy bleeding | L042400 | Complete spontaneous abortion with metabolic disorder |
| L040200 | Unspecified spontaneous abortion + pelvic organ/tissue damage | L042500 | Complete spontaneous abortion with shock |
| L040300 | Unspecified spontaneous abortion with renal failure | L042600 | Complete spontaneous abortion with embolism |
| L040400 | Unspecified spontaneous abortion with metabolic disorder | L042w00 | Complete spontaneous abortion + other specified complication |
| L040500 | Unspecified spontaneous abortion with shock | L042x00 | Complete spontaneous abortion with complication NOS |
| L040600 | Unspecified spontaneous abortion with embolism | L042y00 | Complete spontaneous abortion + no mention of complication |
| L040900 | Inevitable miscarriage | L042z00 | Complete spontaneous abortion NOS |
| L040w00 | Unspecified spontaneous abortion + other specified complication | L043.00 | Inevitable abortion unspecified |
| L040x00 | Unspecified spontaneous abortion with complication NOS | L043.11 | Inevitable miscarriage unspecified |
| L040y00 | Unspecified spontaneous abortion without mention of complication | L043000 | Unspecified inevitable abortion comp by genital tract and pelvic infect |
| L040z00 | Unspecified spontaneous abortion NOS | L043011 | Unspecified inevitable miscarriage comp by genital tract pelvic infection |
| L041.00 | Spontaneous abortion incomplete | L043100 | Unspecified inevitable abortion comp by delayed or excessive hemorrhage |
| L041000 | Incomplete spontaneous abortion + genital tract/pelvic infection | L043111 | Unspecified inevitable miscarriage comp by delayed or excessive hemorrhage |
| L041100 | Incomplete spontaneous abortion + delayed/excessive hemorrhage | L043200 | Unspecified inevitable abortion complicated by embolism |
| L041200 | Incomplete spontaneous abortion + pelvic organ/tissue damage | L043211 | Unspecified inevitable miscarriage complicated by embolism |
| L041300 | Incomplete spontaneous abortion with renal failure | L043x00 | Unspecified inevitable abortion with unspecified complication |
| L041400 | Incomplete spontaneous abortion with metabolic disorder | L043x11 | Unspecified inevitable miscarriage with unspecified complication |
| L041500 | Incomplete spontaneous abortion with shock | L043y00 | Unspecified inevitable abortion with OS complication |
| L041600 | Incomplete spontaneous abortion with embolism | L043y11 | Unspecified inevitable miscarriage with OS complication |
| L041w00 | Incomplete spontaneous abortion + other specified complication | L043z00 | Unspecified inevitable abortion without complication |
| L041x00 | Incomplete spontaneous abortion with complication NOS | L043z11 | Unspecified inevitable miscarriage without complication |
| L041y00 | Incomplete spontaneous abortion with no mention of complication | L044.00 | Inevitable abortion incomplete |
| L041z00 | Incomplete spontaneous abortion NOS | L045x11 | Complete inevitable miscarriage with unspecified comp |
| L044.00 | Inevitable abortion incomplete | L045y00 | Complete inevitable abortion with OS complication |
| L044.11 | Inevitable miscarriage incomplete | L045y11 | Complete inevitable miscarriage with OS complication |
| L044000 | Incomplete inevitable abortion complicated by genital tract and pelvic infection | L045z00 | Complete inevitable abortion without complication |
| L044011 | Incomplete inevitable miscarriage complicated by genital tract pelvic infect | L045z11 | Complete inevitable miscarriage without complication |
| L044100 | Incomplete inevitable abortion complicated by delayed or excessive hemorrhage | L04z.00 | Spontaneous abortion NOS |
| L044111 | Incomplete inevitable miscarriage comp by delayed or excessive hemorrhage | L02..11 | Missed miscarriage |
| L044200 | Incomplete inevitable abortion complicated by embolism | L02..12 | Silent miscarriage |
| L044211 | Incomplete inevitable abortion complicated by embolism | L045x00 | Complete inevitable abortion with unspecified complication |
| L044x00 | Incomplete inevitable abortion with unspecified complication |  |  |
| L044x11 | Incomplete inevitable miscarriage with unspecified complication |  |  |
| L044y00 | Incomplete inevitable abortion with OS complication |  |  |
| L044y11 | Incomplete inevitable miscarriage with other specified complication |  |  |
| L044z00 | Incomplete inevitable abortion without complication |  |  |
| L044z11 | Incomplete inevitable miscarriage without complication |  |  |
| L045.00 | Inevitable abortion complete |  |  |
| L045.11 | Inevitable miscarriage complete |  |  |
| L045000 | Complete inevitable abortion complicated by genital tract and pelvic infection |  |  |
| L045011 | Complete inevitable miscarriage complicated by genital tract and pelvic infection |  |  |
| L045100 | Complete inevitable abortion comp by delayed or excessive hemorrhage |  |  |
| L045111 | Complete inevitable miscarriage comp by delayed or excessive hemorrhage |  |  |
| L045200 | Complete inevitable abortion complicated by embolism |  |  |
| L045211 | Complete inevitable miscarriage complicated by embolism |  |  |

**Supplemental references**

1. Minassian C, Williams R, Meeraus WH, Smeeth L, Campbell OMR, Thomas SL. Methods to generate and validate a Pregnancy Register in the UK Clinical Practice Research Datalink primary care database. Pharmacoepidemiol Drug Saf. 2019 Jul 1;28(7):923–33.
2. caliberresearch.org. CALIBER [Internet]. [cited 2020 Mar 14]. Available from: https://caliberresearch.org/portal/codelists
